# Genome-Wide Screen Reveals *sec21* Mutants of *Saccharomyces cerevisiae* Are Methotrexate-Resistant

**DOI:** 10.1534/g3.116.038117

**Published:** 2017-02-22

**Authors:** Lai H. Wong, Stephane Flibotte, Sunita Sinha, Jennifer Chiang, Guri Giaever, Corey Nislow

**Affiliations:** * Department of Pharmaceutical Sciences, University of British Columbia, Vancouver, British Columbia V6T 1Z3, Canada; † Department of Zoology, University of British Columbia, Vancouver, British Columbia V6T 1Z3, Canada

**Keywords:** budding yeast, COPI complex

## Abstract

Drug resistance is a consequence of how most modern medicines work. Drugs exert pressure on cells that causes death or the evolution of resistance. Indeed, highly specific drugs are rendered ineffective by a single DNA mutation. In this study, we apply the drug methotrexate, which is widely used in cancer and rheumatoid arthritis, and perform evolution experiments on Baker’s yeast to ask the different ways in which cells become drug resistant. Because of the conserved nature of biological pathways between yeast and man, our results can inform how the same mechanism may operate to render human cells resistant to treatment. Exposure of cells to small molecules and drug therapies imposes a strong selective pressure. As a result, cells rapidly acquire mutations in order to survive. These include resistant variants of the drug target as well as those that modulate drug transport and detoxification. To systematically explore how cells acquire drug resistance in an unbiased manner, rapid cost-effective approaches are required. Methotrexate, as one of the first rationally designed anticancer drugs, has served as a prototypic example of such acquired resistance. Known methotrexate resistance mechanisms include mutations that increase expression of the dihydrofolate reductase (DHFR) target as well as those that maintain function yet reduce the drug’s binding affinity. Recent evidence suggests that target-independent, epistatic mutations can also result in resistance to methotrexate. Currently, however, the relative contribution of such unlinked resistance mutations is not well understood. To address this issue, we took advantage of *Saccharomyces cerevisiae* as a model eukaryotic system that combined with whole-genome sequencing and a rapid screening methodology, allowed the identification of causative mutations that modulate resistance to methotrexate. We found a recurrent missense mutation in *SEC21* (orthologous to human COPG1), which we confirmed in 10 *de novo* methotrexate-resistant strains. This *sec21* allele (S96L) behaves as a recessive, gain-of-function allele, conferring methotrexate resistance that is abrogated by the presence of a wild-type copy of *SEC21*. These observations indicate that the Sec21p/COPI transport complex has previously uncharacterized roles in modulating methotrexate stress.

An unintended consequence of the widespread use of chemotherapy and targeted drugs is the inevitable development of resistance. Drug resistance induced by such personalized therapies evolves in diverse ways. Host factors and genetic changes are well-established contributors that enable cells to evolve clinically significant resistance (Gottesman 2002; Garraway and Janne 2012; Holohan *et al.* 2013). A common mechanism of acquired resistance is to limit drug uptake and/or enhance cellular efflux. The multidrug transporter genes (of the ATP-binding cassette family) play important roles in this multidrug resistance phenomenon (Vasiliou *et al.* 2009). Mutations that alter drug pharmacokinetics and drug-detoxifying mechanisms also facilitate the evolution of resistance (Gottesman 2002; Mikkelsen *et al.* 2011).

It is well established that genetic variation in a drug’s target arises as a direct consequence of treatment (Wacker *et al.* 2012; Palmer and Kishony 2013). Mutations in protein targets have rendered many first-line anticancer therapies ineffective, which is exacerbated by the fact that many of these therapies are required chronically. One such widely studied example is the antifolate drug methotrexate (MTX). MTX is one of the first examples of rational drug design based on the observation that folic acid stimulated the proliferation of acute lymphoblastic leukemia cells; as such, MTX was designed as an antimetabolite to block the folic acid biosynthesis pathway (for review see Farber *et al.* 1948; Mikkelsen *et al.* 2011). Dihydrofolate reductase (DHFR) is a conserved enzyme in the folic acid biosynthesis pathway which is required for the *de novo* synthesis of purines, thymidylic acid, and amino acids (Chen *et al.* 1984; Barclay *et al.* 1988). MTX has been successfully used to treat a wide range of cancers, *e.g.*, non-Hodgkin lymphoma, osteosarcoma, and colon cancer. MTX therapy has, however, led cancer cells to evolve various MTX resistance mechanisms, which include mutations that increase production of the DHFR drug target as well as those that reduce the ability of the drug to bind the target (Lewis *et al.* 1995; Zhao and Goldman 2003; Assaraf 2007; Mikkelsen *et al.* 2011; Wong *et al.* 2016). MTX is also used in other applications, most notably for autoimmune treatment in alleviating the symptoms of rheumatoid arthritis and Crohn’s disease. In these cases, its efficacy is, however, less well understood on a mechanistic level (Mikkelsen *et al.* 2011) and little is known about the development of resistance during these treatments.

There is thus a compelling need to understand the pathways that are affected by MTX treatment and to define the distinct ways in which cells can become resistant to treatment. For example, evidence suggests that target-independent epistatic mutations can contribute to antifolate resistance (Bohanec Grabar *et al.* 2008; Constanzo and Hartl 2011; Toprak *et al.* 2012). Yet the roles of such target-independent resistance mechanisms are poorly understood either qualitatively or quantitatively which can limit new applications of known drugs, in the case of drug-repurposing efforts. A powerful way to address this challenge is to take an unbiased approach and design an experiment in which the cell reports how methotrexate resistance develops. Toward this end, we used *Saccharomyces cerevisiae*, a validated eukaryotic model of MTX activity (Cardenas *et al.* 1999; Hoon *et al.* 2008; Wong *et al.* 2016). Here, we present the results of a resistance screen in wild-type yeast cells to isolate *de novo* yeast mutants conferring resistance to MTX. Whole-genome sequencing analysis of 10 independent mutants confirmed the emergence of a common *sec21* allele (S96L) capable of modulating MTX resistance. The role of *SEC21* was further demonstrated by restoration of MTX sensitivity by introduction of wild-type *SEC21* by transformation and mating.

## Materials and Methods

### Yeast strains and growth conditions

For wild-type reference strains, diploid BY4743 *MATa/MATα his3Δ1/his3Δ1 leu2Δ0/leu2Δ0 LYS2/lys2Δ0 met15Δ0/MET15Δ0 ura3Δ0/ura3Δ0* and haploid BY4742 *MAT*α (*his3*Δ*1 leu2*Δ*0 lys2*Δ*0 ura3*Δ*0*) were used in this study. All growth assays were performed using rich media (yeast extract, peptone, dextrose medium, YPD), synthetic complete (SC) media, or synthetic dropout minus uracil (SD-URA). To verify ρ^0^, cells which lack mitochondrial genome, we tested for growth in obligate respiratory media by culturing cells in YP media with a nonfermentable carbon source (glycerol at 3% v/v). To generate strains in the diploid background, haploid strains were mated to the haploid BY4742 and diploids selected on media lacking lysine and methionine (SD-LYS-MET).

### Drug screen

A methotrexate (MTX) resistance assay was performed by plating wild-type BY4741 yeast cells at a density of 6 × 10^4^ cells onto three 100-mm^2^ petri dishes. Each dish contained YPD agar medium with MTX (Sigma M9929) dissolved at a final 1.2 mM, which is equivalent to a minimum effective concentration that inhibits 20% cell growth (MEC_20_), based on previously published growth assays (Wong *et al.* 2016). An aliquot of the untreated parent BY4741 strain was archived for subsequent validation experiments. Plated cells were cultured for 7 d (until robust colonies were visible) at 30° in the dark. Of 32 colonies recovered, 10 were confirmed to be drug resistant by exposing the individual colonies to fresh MTX-containing YPD agar media. To further validate the growth fitness of candidate MTX-resistant strains, a liquid growth assay was conducted in a Tecan plate shaker overnight at 30° with vigorous shaking (200 rpm). Optical density readings (O.D._595_) were collected over 24 hr in four independent growth assays. Significance of growth differences was assessed with a *t*-test between the mutant and BY4741 wild-type strains taking into account multiple testing with the Benjamini Hochberg procedure.

### Whole-genome sequencing and analysis

Whole-genome sequencing was performed on the confirmed MTX-resistant clones and MTX-sensitive wild-type cells. Genomic DNA was extracted from cells using the Gentra Puregene Yeast/Bact. kit (Qiagen) according to the manufacturer’s instructions. Genomic DNA was quantified using Qubit fluorometry and Nextera XT libraries prepared following manufacturer recommendations (Illumina). Paired-end sequencing (2 × 101 nt) was conducted on an Illumina HiSequation 2500 in Rapid Run mode. Raw base call data (bcl) were converted into FastQ format using the bcl2fastq conversion software from Illumina (version 1.8.3, setting – no-eamss).

Sequence read pairs were mapped to the yeast reference genome S288C version R64 (downloaded from Saccharomyces Genome Database [SGD], http://www.yeastgenome.org) using the short-read aligner BWA version 0.7.9 (Li and Durbin 2009). For each sample this resulted in an average sequencing depth ranging from 63× to 144×. Single-nucleotide variants (SNVs) were identified and filtered with the help of the SAMtools toolbox (Li *et al.* 2009). SNVs also present in the parent strain were eliminated from further consideration. Each SNV was annotated with a custom Perl script and gene information downloaded from SGD on January 21, 2014. The read alignments in the regions of interesting candidate SNVs were visually inspected with the IGV viewer (Robinson *et al.* 2011; Thorvaldsdottir *et al.* 2013) for further curation. The presence of the mitochondria genome was inferred by measuring the number of reads properly aligning to the mitochondrial reference sequence divided by the length of the mitochondrial genome, and then normalizing that ratio to the equivalent quantity for the nuclear genome. Only wild-type BY4741 cells have mitochondrial genome at a calculated ratio of 1:5.

### Yeast transformation and complementation assay

To validate the drug resistance of *sec21* variants, all MTX-resistant strains were transformed with a MoBY-*SEC21* plasmid (Ho *et al.* 2009) using a high-efficiency LiAc-based transformation protocol (Gietz and Schiestl 2007). Transformants were grown on YPD medium containing G418 (geneticin) to select for the MoBY-*SEC21* plasmid. Each individual MoBY-*SEC21* transformed strain was exposed to MTX (1 or 2 mM) in liquid growth assays, as described above.

### Data availability

MTX-resistant strains are available upon request. All the fastq files are deposited at the NCBI SRA under the accession number SRP082437.

## Results

### Genome-wide methotrexate resistance screen

**Figure 1:**
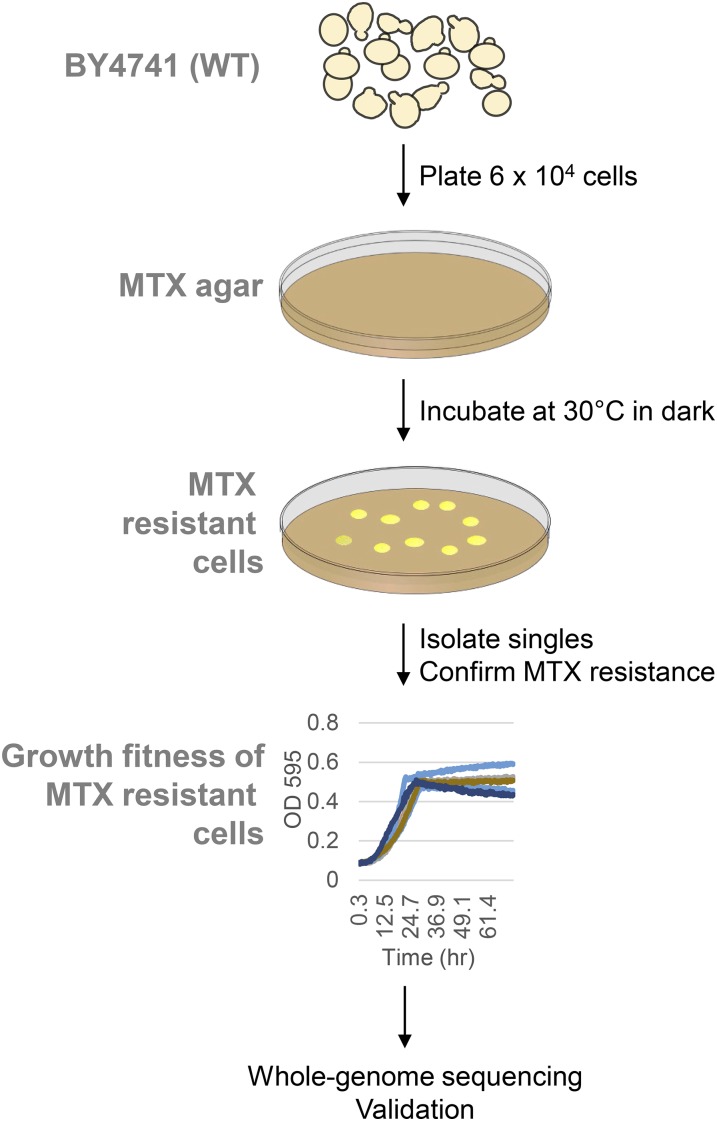
Drug resistance assay. Wild-type BY4741 cells were screened by plating 6 × 10^4^ cells on rich YPD solid media containing methotrexate (1.2 mM, MTX). After 7 d of growth at 30° in the dark, the plate was monitored for the appearance of colonies which were then isolated as single cells. MTX resistance was next independently confirmed by liquid growth assay in the presence of a dose-response of methotrexate. The genomic DNA of the confirmed MTX-resistant colonies was extracted for next-generation sequencing followed by gene variant analysis. Finally genomic alterations identified were validated by complementation assays.

**Figure 2:**
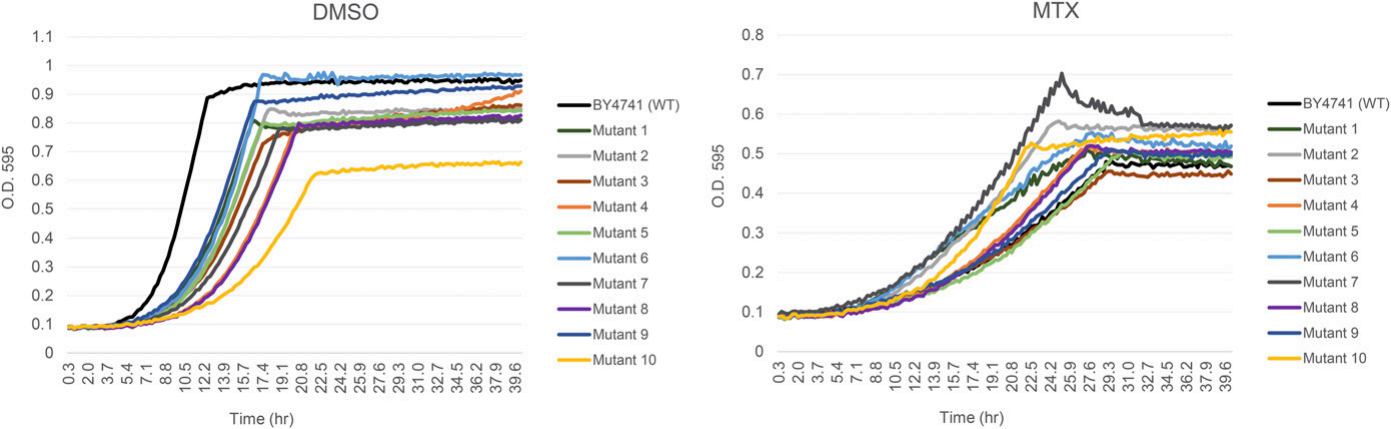
Confirmation of methotrexate resistance in the isolated yeast mutants. Microcultures (100 μl) of wild-type BY4741 (WT, black) and yeast mutants (1–10) grown in YPD media with dimethyl sulfoxide (DMSO) (1% v/v) or methotrexate (MTX, 2 mM) were evaluated by O.D._595_ measurements taken every 15 min over 40 hr in a Tecan shaker-reader (200 rpm) at 30°. Drug resistance was reproduced in two independent liquid growth assays.

**Figure 3:**
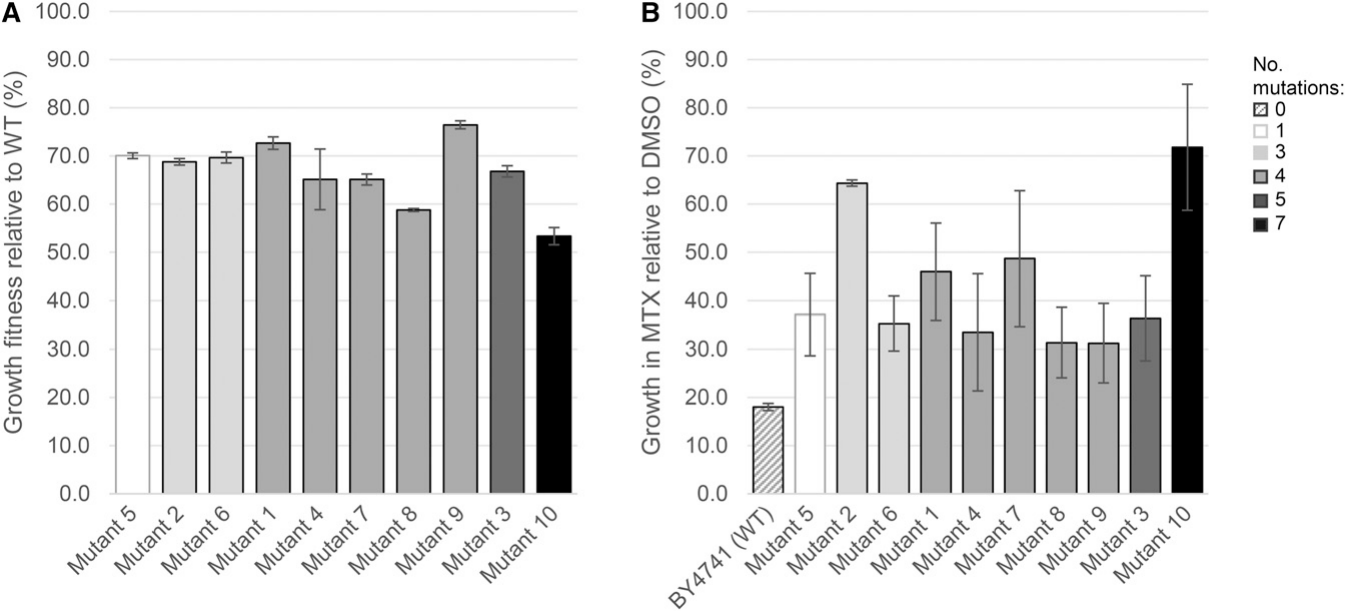
Methotrexate resistance and fitness assayed by liquid growth assay. The growth of yeast mutants in YPD media supplemented with MTX (2 mM) or DMSO solvent (1% v/v) is shown. Microcultures (100 μl) were evaluated by O.D._595_ measurements taken every 15 min over 24 hr in a Tecan shaker-reader (200 rpm) at 30°. Yeast mutant strains (1–10) are color coded (gray gradient) by the number of synonymous and nonsynonymous mutations detected (1, 3, 4, 5, or 7) and compared to the methotrexate-sensitive BY4741 strain (WT, hashed) (Table S1). (A) The doubling time of methotrexate-resistant mutants grown in YPD media containing DMSO (1% v/v) relative to the wild-type BY4741 strain was compared. Error bars indicate SE, n = 3. (B) The growth (expressed as doubling time) of yeast mutants cultured in MTX-containing YPD media relative to DMSO solvent control is evaluated. Error bars indicate SE, n = 4. The growth rate (hours) of mutants 2 and 10 are statistically significant with respect to the wild-type strain, after taking into account multiple testing (p values of 0.005 and 0.002 for mutants 2 and 10, respectively) (see *Materials and Methods*). Error bars indicate SE, *n* = 3.

To isolate MTX-resistant mutants, 6 × 10^4^ wild-type cells were plated in three agar plates with lethal doses of MTX and allowed to recover at 30° in the dark (Figure 1). Seven days post-treatment, 32 robust colonies had formed. Ten of these 32 were confirmed as *bona fide* resistant mutants by growth in media containing MTX (Figure 2 and *Materials and Methods*). These MTX-resistant mutants showed a range of dose-dependent growth in high doses of MTX (MEC_100_ 2 mM, Wong *et al.* 2016), and furthermore, their competitive fitness varied (Figure 2 and Figure 3B). In the absence of drug challenge, however, all mutants exhibited growth defects relative to the wild type (Figure 2 and Figure 3A). This observation that each mutant’s fitness was distinct either in the presence or absence of MTX prompted us to determine the whole-genome sequence of each mutant.

### Genomic variations underlying methotrexate drug resistance

Whole-genome sequencing was performed for each independent MTX-resistant mutant (see *Materials and Methods*). These data identified a common *sec21* missense mutation (S96L) that results in the substitution of serine with leucine at position 96 in all 10 mutants (Table 1 and Supplemental Material, Table S1). This recurrent variant strongly suggests that, despite the fact that each of these 10 mutants had other polymorphisms, the *sec21*(S96L) is a “driver” mutation that resulted in the MTX-resistant phenotype. In addition to the *sec21* allele, all mutants were ρ^0^ and devoid of mitochondrial DNA (Table S1). Consistent with this observation, none of these mutants was able to grow in obligate respiratory YP media containing glycerol (data not shown). Inhibition of folic acid biosynthesis, which is essential for DNA synthesis, is known to hinder mitochondrial DNA replication and consequently lead to respiration deficiency (Wintersberger and Hirsch 1973; Blount *et al.* 1997; Stover and Field 2011). However, respiration deficiency has not been associated specifically with MTX resistance. These observations suggest that, because mitochondrial activity is dispensable for *S. cerevisiae* growth in fermentation conditions (Yotsuyanagi 1962; Otterstedt *et al.* 2004), these mutants can survive in response to MTX at the expense of overall fitness and loss of mitochondrial DNA. Further, our inability to uncover *dfr1* mutants is consistent with the fact that we screened haploid yeast (*Materials and Methods*). Because Dfr1p is an essential enzyme, mutations that compromise MTX drug binding, which are known to abolish the catalytic activity of Dfr1p, would likely be inviable (Wong *et al.* 2016).

**Table 1:** Common genomic variation in 10 methotrexate-resistant strains

Group	Sample ID	Genomic Variants
A	2; 3; 5; 6; 9	*sec21*(S96L); ρ^0^
B	4; 7; 8	*sec21*(S96L); ρ^0^; *vas*1(F227V)
C	1; 10	*sec21*(S96L); ρ^0^; *dit1*(A39T); *fkh1*(A443D); *rkm1*(Y418N)

Genomic variants shared between resistant strains are binned into groups A, B, and C. Unlike the parent BY4741 strain, all mutant strains are ρ^0^, lacking mitochondrial genome.

In addition to the *sec21*(S96L) allele, three out of the 10 mutants (4, 7, and 8) carry a common missense mutation in the *VAS1* gene (F227V); two mutants (1 and 10) had three other nonsynonymous mutations: *dit1*(A39T); *fkh1*(A443D); *rkm1*(Y418N) (Table 1 and Table S1). Since *DIT1*, *FKH1*, and *RKM1* gene functions are required for lifespan and survival (Briza *et al.* 1990; Porras-Yakushi *et al.* 2005; Postnikoff *et al.* 2012), it is perhaps not surprising that these later two MTX-resistant strains, in particular mutant 10, which carry four independent missense mutations and three additional genomic polymorphisms, manifest the lowest overall fitness (Figure 3B and Table S1).

### SEC21 complementation of methotrexate sensitivity

**Figure 4:**
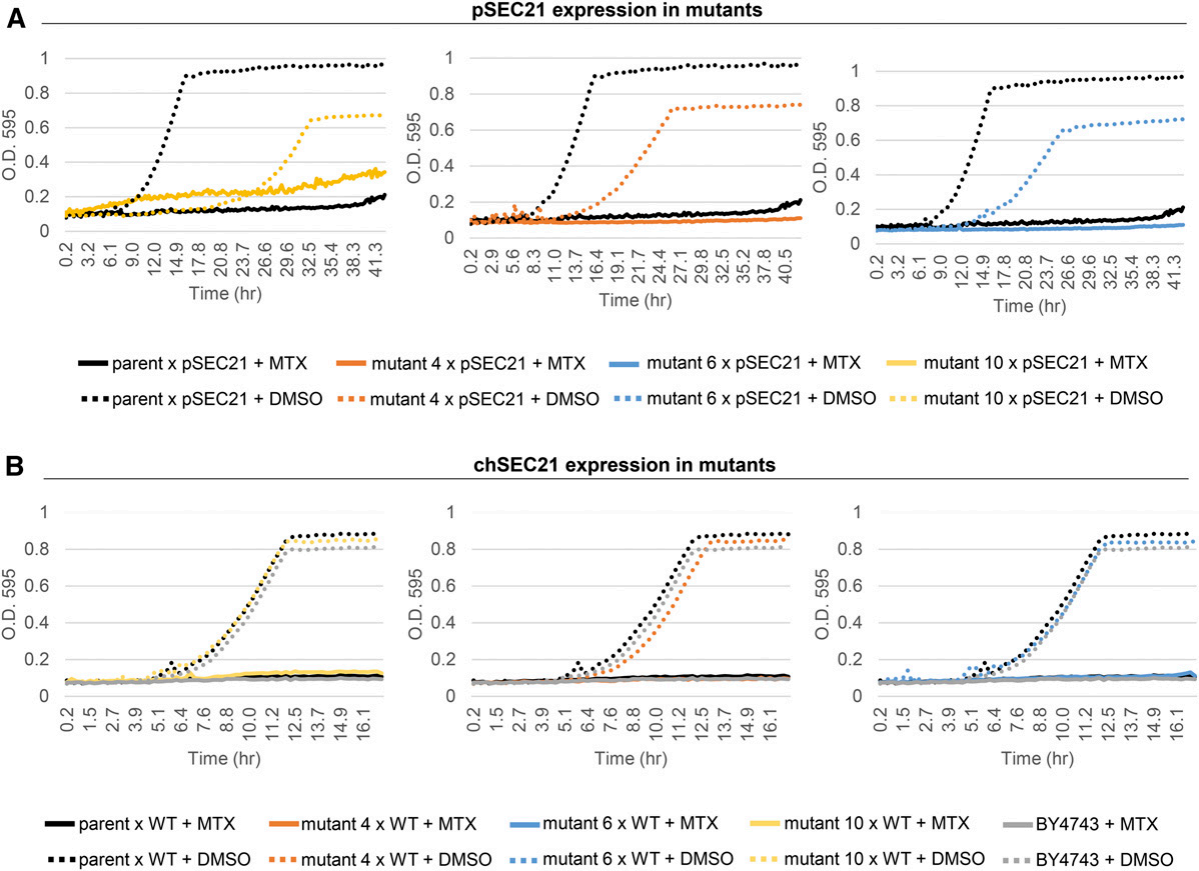
*SEC21* complementation assays. As in Figure 1, growth of microcultures (100 μl) was evaluated by O.D._595_ measurements taken every 15 min over time in a Tecan shaker-reader (200 rpm) at 30°. (A) The effect of *SEC21* expression (MoBY-*SEC21* plasmid, pSEC21) on the fitness of representative methotrexate (MTX)-resistant yeast mutants (4, 6, 10) and the MTX-sensitive parent strain (parent) upon exposure to MTX-containing SD-URA media (1 mM) or DMSO solvent (1% v/v). (B) As in A, the fitness of representative MTX-resistant *sec21/SEC21* heterozygous mutants (containing one wild-type chromosomal copy of *SEC21* – chSEC21) and WT mitochondrial genomes was evaluated (see *Materials and Methods*). Diploid yeast strains were grown in SC media containing MTX (1 mM) or DMSO (1% v/v). Wild-type diploid strain (BY4743) was included as an MTX-sensitive control. Complementation assays were reproduced in three independent experiments.

**Figure 5:**
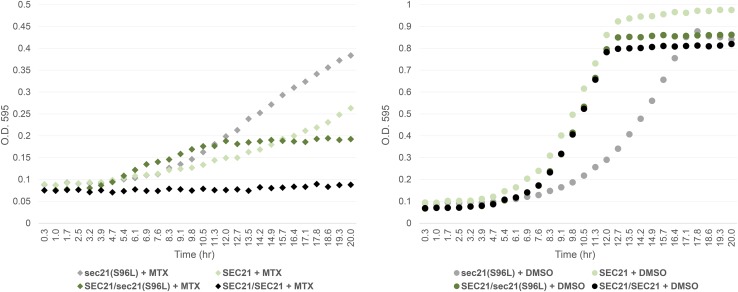
The effect of *SEC21* gene dosage in methotrexate-containing media. As in Figure 1, growth of yeast strains, BY4741 (*SEC21*, in light green), haploid mutant 6 [*sec21*(S96L), in gray], BY4743 (*SEC21*/*SEC21*, in black), and heterozygote mutant 6 [*SEC21*/*sec21*(S96L), in dark green], was evaluated over time in a Tecan shaker-reader (200 rpm) at 30°. Yeast strains were grown in rich media containing MTX (1 mM, diamond markers) and DMSO (1% v/v, circle markers). Growth assays were reproduced in three independent experiments.

Given that the same *sec21*(S96L) allele was found in all MTX-resistant mutants, we conducted complementation assays using wild-type *SEC21*. Specifically, we asked if introduction of a wild-type copy of the essential gene *SEC21* on a plasmid (driven by its native promoter) or by generating a *SEC21*/*sec21*(S96L) heterozygous diploid by mating was sufficient to restore MTX sensitivity (see *Materials and Methods*). When *SEC21* was expressed in each mutant from a MoBY-*SEC21* plasmid (Ho *et al.* 2009) (Figure 4A) or by crossing to a wild-type haploid BY4742 strain (Figure 4B), in both cases, the resistant mutants were resensitized to MTX. While both strategies restored MTX sensitivity, only the heterozygotes showed wild-type fitness in the absence of MTX. Given that only the heterozygotes contained a functional mitochondrial genome, we speculate that only ρ^+^ cells are able to manifest wild-type fitness (Figure 4B). These observations suggest that *sec21*(S96L) acts as a recessive, neomorphic allele to confer MTX resistance. To further support this conclusion, we also found that the greatest degree of MTX sensitivity was achieved when two copies of wild-type *SEC21* were expressed in cells (Figure 5). These are consistent with *SEC21* representing an indirect target of MTX that nonetheless can confer robust drug resistance in a gene dosage-dependent manner, similarly to *DFR1* target (Yan *et al.* 2009).

## Discussion

In the current work, we extend the utility of budding yeast as a model system to study drug resistance by identifying and validating *de novo* mutations capable of conferring resistance to the antifolate drug MTX. Using a growth-based resistance screen combined with whole-genome sequencing, we confirmed that a single “driver” mutation in *SEC21*(S96L) (orthologous to the human COPG1) of the COPI coatomer protein complex (Hosobuchi *et al.* 1992) was associated with previously unknown antifolate tolerance. The protein sequences of yeast Sec21p and human ortholog COPG1 are poorly conserved (32% identical), and in both cases, the yeast S96 and human A96 are located at the surface of the protein and have no annotated function (PDB ID 5A1U). Phenotypically, however, this nonsynonymous mutation, *sec21*(S96L), manifests a gain of MTX resistance phenotype that can be suppressed by the presence of a wild-type copy of *SEC21* in a gene dosage-dependent manner (Figure 4 and Figure 5). In light of our complementation results, we asked if *SEC21* haploinsufficiency can result in MTX-induced growth defects. In fact, in our previously published compendium of chemogenomic profiles (Lee *et al.* 2014), *sec21*Δ/*SEC21* heterozygotes showed a statistically significant MTX-induced growth sensitivity. Together, these findings suggest that *sec21*(S96L) mutants resemble *SEC21*/*sec21*Δ cells – in both cases cells show increased MTX resistance; and *sec21*(S96L) mutants behave like partial loss-of-function alleles – in this scenario *SEC21* function is required to resist MTX. Further analysis showed that deletion of five out of seven genes in the coatomer complex, specifically *SEC26* (human COPB1), *SEC27* (human COPB2), *RET2* (human ARCN1), COP1 (human COPA), and *RET3* (human COPZ), sensitizes yeast cells to the presence of MTX (Lee *et al.* 2014). The latter results suggest that these coatomer subunits, which are required for coatomer biogenesis, integrity, and assembly of COP-coated vesicles (Kuge *et al.* 1993; Beck *et al.* 2009), may be important in mediating the transport of proteins implicated in MTX metabolism and associated metabolic effects that may influence MTX treatment response. In fact, MTX therapy has also been used to alleviate symptoms of arthritis in patients who suffer from COPA syndrome, an immune dysregulatory disease caused by defective COPA gene variants (Vece *et al.* 2016). These observations reveal that the COPI transport complex may have previously uncharacterized roles in modulating methotrexate stress.

Since MTX resistance can be abrogated by the expression of wild-type *SEC21* alone, the additional rare mutations acquired by MTX-resistant mutants are likely to be nonconsequential with respect to the phenotype, which have emerged as a result of dynamic survival strategies cells evolved to respond to drug selection (Table S1). Upon MTX-induced mitochondrial damage, it is not surprising to find additional polymorphisms in genes such as *VAS1* and *DIT1* that can further impair mitochondria homeostasis, which in turn help cells to eliminate dysfunctional mitochondria to guarantee cell survival (Chatton *et al.* 1988; Briza *et al.* 1990; de la Torre-Ruiz *et al.* 2015). Particularly, three MTX-resistant mutants with reduced survival fitness emerged with a common *vas1*(F227V) variant, which potentially disrupts the mitochondrial DNA synthesis given Vasp1 function as an essential mitochondrial valyl-tRNA synthetase (Chatton *et al.* 1988). Furthermore, *rkm1*(Y418N) and *fkh1*(A443D) mutations, which can alter chromatin state and cell cycle progression (Porras-Yakushi *et al.* 2005; Postnikoff *et al.* 2012), could potentially facilitate the long-term propagation of MTX-resistant cells. Functional studies to further understand the putative effects of these rare mutations will help to uncover the underlying defense mechanisms implicated in antifolate-tolerant cell survival.

Together, these findings suggest that the conserved function of COPI coatomer complex in the intercompartmental vesicle-mediated transport regulation (Duden *et al.* 1994; Gaynor *et al.* 1998; Beck *et al.* 2009), plays a key role for the metabolic trafficking of detoxification proteins associated with MTX metabolism and clearance (Stover and Field 2011). Disruption of the COPI complex may therefore represent a novel cellular pathway to regulate antifolate stress that could ultimately improve antifolate regimes with more tolerable toxicity profiles. Further understanding of the relationship between the secretory pathways, COPI complex, and antifolate metabolism should guide the implementation of better therapeutic strategies to modulate the emergence of drug-resistant variants.

## Supplementary Material

Supplemental material is available online at www.g3journal.org/lookup/suppl/doi:10.1534/g3.116.038117/-/DC1.
