# Supplementary material for: Genome-Wide Screen Reveals sec21 Mutants of Saccharomyces cerevisiae Are Methotrexate-Resistant
Source: G3 (Bethesda). 2017 Feb 22;7(4):1251–7. doi: 10.1534/g3.116.038117 (PMC5386873; doi:10.1534/g3.116.038117)
Supplement: Supplementary file 1 [file 1251file001.docx]

**Supplementary Material**

**Supplementary Table 1** Variant calls in methotrexate resistant yeast strains. For each mutant strain (IDs 1-10) assigned with a sequencing sample ID name (EWpar1-EWpar10), all genomic alterations identified are listed. The mitochondrial genome (chrMito) is absent in all 10 mutant strains.

| Sample ID | Strain ID | Chr | Coordinate | Locus | Gene | Mutation | Variant | Residue |
| --- | --- | --- | --- | --- | --- | --- | --- | --- |
| EWpar1 | 1 | chrIV | 1274716 | CDS | DIT1 | missense | 115G>A | A39T |
| EWpar1 | 1 | chrIX | 100908 | CDS | FKH1 | missense | 1328G>T | A443D |
| EWpar1 | 1 | chrXIV | 92280 | CDS | SEC21 | missense | 287C>T | S96L |
| EWpar1 | 1 | chrXVI | 159093 | CDS | RKM1 | missense | 1252T>A | Y418N |
| EWpar1 | 1 | chrMito | - | - | - | absent | - | - |
| EWpar2 | 2 | chrIV | 620955 | ncRNA | - | - | - | - |
| EWpar2 | 2 | chrIV | 1225687 | CDS | BCS1 | synonymous | 850A>G | L284L |
| EWpar2 | 2 | chrXIV | 92280 | CDS | SEC21 | missense | 287C>T | S96L |
| EWpar2 | 2 | chrMito | - | - | - | absent | - | - |
| EWpar3 | 3 | chrIV | 916529 | CDS | ADK1 | missense | 44C>T | P15L |
| EWpar3 | 3 | chrV | 396712 | promoter | RPL23B | - | -57G>C | - |
| EWpar3 | 3 | chrVII | 702810 | CDS | YGR107W | synonymous | 144G>T | P48P |
| EWpar3 | 3 | chrVII | 881830 | CDS | HIP1 | missense | 1411C>A | H471N |
| EWpar3 | 3 | chrXIV | 92280 | CDS | SEC21 | missense | 287C>T | S96L |
| EWpar3 | 3 | chrMito | - | - | - | absent | - | - |
| EWpar4 | 4 | chrVII | 672864 | CDS | VAS1 | missense | 679T>G | F227V |
| EWpar4 | 4 | chrXI | 548303 | CDS | RHO4 | missense | 88C>A | L30I |
| EWpar4 | 4 | chrXIV | 92280 | CDS | SEC21 | missense | 287C>T | S96L |
| EWpar4 | 4 | chrXV | 817715 | CDS | YOR262W | missense | 424G>A | D142N |
| EWpar4 | 4 | chrMito | - | - | - | absent | - | - |
| EWpar5 | 5 | chrXIV | 92280 | CDS | SEC21 | missense | 287C>T | S96L |
| EWpar6 | 6 | chrXII | 314195 | CDS | CSF1 | missense | 1537G>A | P513S |
| EWpar6 | 6 | chrXIV | 92280 | CDS | SEC21 | missense | 287C>T | S96L |
| EWpar6 | 6 | chrXIV | 148631 | CDS | POL2 | missense | 420T>A | F140L |
| EWpar6 | 6 | chrMito | - | - | - | absent | - | - |
| EWpar7 | 7 | chrII | 739991 | CDS | REI1 | missense | 151G>C | D51H |
| EWpar7 | 7 | chrVII | 672864 | CDS | VAS1 | missense | 679T>G | F227V |
| EWpar7 | 7 | chrXI | 232006 | intergenic | - | - | - | - |
| EWpar7 | 7 | chrXIV | 92280 | CDS | SEC21 | missense | 287C>T | S96L |
| EWpar7 | 7 | chrMito | - | - | - | absent | - | - |
| EWpar8 | 8 | chrVII | 672864 | CDS | VAS1 | missense | 679T>G | F227V |
| EWpar8 | 8 | chrXIV | 92280 | CDS | SEC21 | missense | 287C>T | S96L |
| EWpar8 | 8 | chrXIV | 227412 | CDS | ATG4 | missense | 43A>G | K15E |
| EWpar8 | 8 | chrXV | 973811 | CDS | YOR343W-B | missense | 1939A>G | N647S |
| EWpar8 | 8 | chrMito | - | - | - | absent | - | - |
| EWpar9 | 9 | chrI | 110707 | 3’- UTR | CCR4 | - | -139G>A | - |
| EWpar9 | 9 | chrIV | 1331198 | intergenic | - | - | - | - |
| EWpar9 | 9 | chrVII | 56346 | promoter | MTO1 | - | -550G>A | - |
| EWpar9 | 9 | chrXIV | 92280 | CDS | SEC21 | missense | 287C>T | S96L |
| EWpar9 | 9 | chrMito | - | - | - | absent | - | - |
| EWpar10 | 10 | chrIV | 1274716 | CDS | DIT1 | missense | 115G>A | A39T |
| EWpar10 | 10 | chrIX | 100908 | CDS | FKH1 | missense | 1328G>T | A443D |
| EWpar10 | 10 | chrX | 745667 | telomere | - | - | - | - |
| EWpar10 | 10 | chrXIII | 378784 | ncRNA | - | - | - | - |
| EWpar10 | 10 | chrXIV | 92280 | CDS | SEC21 | missense | 287C>T | S96L |
| EWpar10 | 10 | chrXVI | 67646 | intergenic | - | - | - | - |
| EWpar10 | 10 | chrXVI | 159093 | CDS | RKM1 | missense | 1252T>A | Y418N |
| EWpar10 | 10 | chrMito | - | - | - | absent | - | - |
